# Catheter-associated bloodstream infections in an ICU of a university hospital in Wroclaw, Poland: an international nosocomial infection control consortium's findings

**DOI:** 10.1186/cc14154

**Published:** 2015-03-16

**Authors:** W Duszynska, VD Rosenthal, A Litwin, E Woznica, A Kubler

**Affiliations:** 1 University Hospital, Wroclaw, Poland; 2 International Nosocomial Infection Control Consortium, Buenos Aires, Argentina

## Introduction

Catheter-associated bloodstream infections are serious but potentially possible to reduce complication of treatment in the ICU. The aim of the study was to evaluate the frequency and etiology of central line-associated bloodstream infections (CLA-BSI) in ICU patients according to the International Nosocomial Infection Control Consortium (INICC) project.

## Methods

A prospective, observational study was conducted in the 20-bed ICU of the University Hospital in Wroclaw from January 2011 to November 2014. CLA-BSI were diagnosed and evaluated according to protocols standardized by the INICC. The density of CLA-BSI/1,000 central line-days, the incidence index/100 admissions to the hospital, the central line utilization ratio (CL-UR) as well as the microbiological profile of CLA-BSI were evaluated. The results were compared with our earlier published data and with the findings of international reports.

## Results

Among 1,746 ICU patients, CLA-BSI were diagnosed in 69 cases. The incidence index was 3.88/100 admissions to the ICU.CLA-BSI were diagnosed in 18% of the overall number (381) of device-associated healthcare-associated infections. Central line was used in 91.41 ± 4.4% patients during 19,819 patient-days and 18,155 central line-days. The median density of CLA-BSI/1,000 central line-days was 3.88/3.77/3.36 and 0.0 accordingly in years 2011/2012/2013 and 2014 (from January to November). The main pathogens of CLA-BSI were CN staphylococci (22%), *Staphylococcus aureus *(21%), and Enterobacteriaceae (29%). In this study, the density of CLA-BSI was about 50% lower (2.75 (2.0 to 6.06)) than in our previous study and in the INICC's report (2014), but higher than in the CDC's NHSN (2012) report.

## Conclusion

The implementation of the infection control program and preventive interventions for patients with central venous catheters improved the safety and quality of healthcare in the ICU by reducing CLA-BSI rate.
